# Mode of delivery in non-cephalic presenting twins: a systematic review

**DOI:** 10.1007/s00404-012-2294-6

**Published:** 2012-04-01

**Authors:** Charlotte N. Steins Bisschop, Tatjana E. Vogelvang, Anne M. May, Nico W. E. Schuitemaker

**Affiliations:** 1 Julius Center for Health Sciences and Primary Care, University Medical Center Utrecht, Str. 6.131, P.O. Box 85500, 3508 GA Utrecht, The Netherlands; 2 Department of Obstetrics and Gynecology, Diakonessenhuis, Utrecht, The Netherlands

**Keywords:** Twins, Non-cephalic presentation, Mode of delivery, Systematic review

## Abstract

**Purpose:**

This systematic review aims to determine if there are evidence-based recommendations for the optimal mode of delivery for non-cephalic presenting first- and/or second twins. We investigated the impact of the mode of delivery on neonatal outcome for twin deliveries with (1) the first twin (twin A) in non-cephalic presentation, (2) the second (twin B) in non-cephalic presentation and (3) both twins in non-cephalic presentation.

**Methods:**

A computer-aided search of Medline, Embase, Cinahl and Cochrane databases was carried out and quality of the studies was assessed with the Cochrane Collaboration’s tool for assessing risk of bias and the GRADE approach.

**Results:**

One high-quality clinical trial (60 twin pairs) and 16 moderate/low-quality observational studies (3,167 twin pairs) showed no difference in neonatal outcome between vaginal and caesarean delivery in twin A and/or B.

**Conclusion:**

Our results do not suggest benefit of caesarean over vaginal delivery for selected twin gestations with twin A and/or twin B in non-cephalic presentation. However, no final conclusion can be drawn due to the small sample sizes and statistic limitations of the included studies. Randomized studies with sufficient power are required to make a strong recommendation.

## Introduction

The incidence of twin pregnancy has increased largely because of the proliferation of assisted reproductive technologies and the rise in maternal age [1]. Twin gestations comprise approximately 1 % of all pregnancies but account for nearly 10 % of perinatal mortality [2, 3]. The increased morbidity and mortality of twin gestations is frequently attributed to preterm birth, intrauterine growth restriction and other unique complications of twin gestations such as twin–twin transfusion syndrome [4]. Hazards of twin delivery can be attributed to non-cephalic presentation as well [5]. Non-cephalic presentation of the first twin (twin A), the second twin (twin B) or both twins occurs in about 60 % of all twin pregnancies [2, 4, 5].

No consensus about the appropriate mode of delivery for non-cephalic presenting twins exists [6, 7]. Neither the practice bulletin on multiple gestation of the American College of Obstetricians and Gynecologists (ACOG) nor the guideline on multiple gestation of the Dutch Society for Obstetrics and Gynecology (NVOG) makes a recommendation for their route of delivery [6, 7]. Additionally, there is a general uncertainty about vaginal delivery of non-cephalic presenting twins, which is reflected by an increasing number of caesarean deliveries in twin gestations. In the United States, in 2003, 67 % of all twins were delivered by a caesarean section. Some obstetricians cite ‘twins’ as their only indication [8]. A policy of planned caesarean section might increase the risk of neonatal and maternal complications, like neonatal respiratory problems [2] or maternal febrile morbidity [9].

This systematic review aims to determine if there are evidence-based recommendations for the optimal mode of delivery for non-cephalic presenting first and/or second twins. We will investigate the impact of the mode of delivery on neonatal outcome for twin deliveries with (1) twin A in non-cephalic presentation, (2) twin B in non-cephalic presentation and (3) both twins in non-cephalic presentation.

## Methods

### Search strategy

A computer-aided search of Medline, Embase, Cinahl and Cochrane databases was carried out. The following search terms (with synonyms) were used: ‘twins’, ‘non-cephalic’ and ‘delivery’ (Appendix 1). Reference lists of identified studies were searched for additional relevant studies.

### Inclusion criteria

Studies that compared the neonatal outcome (5-min Apgar scores and neonatal mortality) after vaginal delivery with the neonatal outcome after caesarean delivery for non-cephalic presenting twins were included. Twin A, twin B or both twin(s) had to be in non-cephalic presentation. Data of neonatal outcome had to be presented according to the mode of delivery. The twin pregnancy had to reach at least 32 weeks of gestation and both of the twins had to weigh at least 1,500 g. Every study that was published in English language was considered for inclusion, except review articles, case reports or poster session abstracts.

### Selection of studies

The first reviewer (CN) screened the titles and abstracts of identified studies for eligibility. Papers that seemed to be relevant were obtained, and the full text articles were read for inclusion. If there was doubt about the suitability of the studies, they were discussed with another independent reviewer (TE).

### Quality assessment

The first reviewer (CN) independently assessed various aspects of methodological quality of the included studies without masking the source or authorship of the articles. The Cochrane Collaboration’s tool for assessing risk of bias was used [10]. This tool consists of nine items about selection-, performance-, detection-, attribution- and reporting bias. Furthermore, the included studies were scored according to the GRADE approach [10].

### Data extraction and analysis

Due to the heterogeneity of the data, studies could not be pooled. Therefore, we described per study whether a significant difference between vaginal and caesarean delivery was found in (1) low 5-min Apgar scores (<7) and (2) neonatal mortality. The 5-min Apgar scores <7 are widely used in the literature as measurement for poor neonatal outcome [2, 3, 8]. We made a distinction between the neonatal outcome of twin A and twin B. Significant differences were defined according to the definitions and statistics used in the different studies. We described the studies according to the presentation of the twins, i.e. (1) twin A in non-cephalic presentation, (2) twin B in non-cephalic presentation and (3) both twins in non-cephalic presentation.

## Results

**Fig. 1:**
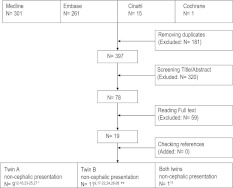
Literature search. Search updated September 18th 2011. *N* number of articles.* Single asterisk* indicates that one study was published in two articles [14, 15].* Double asterisks* indicate that two articles [24, 27] included one subgroup with twin A and one subgroup with twin B in non-cephalic presentation

We identified 578 articles. Nineteen articles reporting the results of 18 studies that compared vaginal delivery with caesarean delivery for non-cephalic presenting twins were included [9, 11–28] (Fig. 1).

### Quality assessment (Table 1)

None of the 18 included studies were blinded since blinding for the mode of delivery was not possible for patients, personnel and outcome assessors.

**Table 1:** Quality assessment: risk of bias

Author	Year	Study design	Selection bias		Performance bias	Detection bias	Attribution bias				Reporting bias	Total	GRADE
			Random sequence generation	Allocation concealment	Blinding of participants and personnel	Blinding of out come assessment	Incomplete outcome data 5-min AS <7 twin A	Incomplete outcome data 5-min AS <7 twin B	Incomplete outcome data Neonatal mortality twin A	Incomplete outcome data Neonatal mortality twin B	Selective outcome dataporting	Items ‘high re-risk of bias’	
Rabinovici [9]	1986	RCT	Low	Low	High	High	Low	Low	Low	Low	Low	2	1
Essel [11]^a^	1996	Prosp cohort	High	High	High	High	Low	Low	Low	Low	Unclear	4	2
Sentilhes [12]	2007	Retr cohort	High	High	High	High	Low	Low	Low	Low	Unclear	4	3
Griasaru [13]	2000	Retr cohort	High	High	High	High	Low	Low	Low	Low	Unclear	4	3
Abu-Heija [14, 15]	1998	Retr cohort	High	High	High	High	Low	Low	Low	Low	Unclear	4	3
Blickstein [16]^b^	1993	Retr cohort	High	High	High	High	Low	Low	Low	Low	Unclear	4	3
Wells [17]	1991	Retr cohort	High	High	High	High	Low	Low	Low	Low	Unclear	4	3
Gocke [18]	1989	Retr cohort	High	High	High	High	Low	Low	Low	Low	Unclear	4	3
Caukwell [19]	2002	Retr cohort	High	High	High	High	High	Low	Low	Low	Unclear	5	3
Winn [20]	2001	Retr cohort	High	High	High	High	High	Low	Low	Low	Unclear	5	3
Acker [21]	1981	Retr cohort	High	High	High	High	High	Low	Low	Low	Unclear	5	3
Atis [22]	2011	Retr cohort	High	High	High	High	High	Low	High	Low	Unclear	6	3
Nassar [23]	2004	Retr cohort	High	High	High	High	No	High	Low	High	Unclear	6	3
Roopnarinesingh [24]	2002	Retr cohort	High	High	High	High	Low	High	Low	High	Unclear	6	3
Blickstein [25]	2000	Retr cohort	High	High	High	High	Low	High	Low	High	Unclear	6	3
Mauldin [26]	1998	Prosp cohort	High	High	High	High	Low	Low	High	High	Unclear	6	3
Kelsick [27]	1982	Retr cohort	High	High	High	High	High	High	Low	High	Unclear	7	3
Greig [28]	1992	Retr cohort	High	High	High	High	High	Low	High	High	Unclear	7	3

For quality assessement the Cochrane Collaboration’s tool for assessing risk of bias [10] and the GRADE classification [10] was used

1 = GRADE: high = randomized trials, or double upgraded observational studies

2 = GRADE: moderate = downgraded randomized trials, or upgraded observational studies

3 = GRADE: low = double downgraded randomized trials, or observational studies

4 = GRADE: very low = triple downgraded randomized trials, or downgraded observational studies, or case series/case reports

*RCT* Randomized clinical trial, *Prosp* Prospective, *Retr* Retrospective

^a^Essel [11]: only the abstract was available

^b^Blickstein [16]: only the abstract was available

According to the GRADE classification [10], only one randomized clinical trial was identified which was of high quality [9]. According to the Cochrane Collaboration’s Tool for assessing the risk of bias [10], this trial described adequate methods of randomisation and concealment of allocation. Two out of 33 women randomized for vaginal delivery subsequently underwent caesarean section (one because of inadequate progress of labour and another because of heart rate monitoring of twin B suggesting foetal distress). Neonatal outcome was completely described for both twins in this study.

The remaining moderate- [11] or low-quality [12–28] observational studies reported different completeness of neonatal outcome data for both twins. None of the observational studies provided information about how the possibility of selective outcome reporting was examined.

### Twin A in non-cephalic presentation (Tables 2, 3)

Eight low-quality observational studies including 1,475 twin pairs compared the mode of delivery of twins with the twin A in non-cephalic presentation [12–16, 23–25, 27]. For twin A, none of the eight studies reported a significant difference in low 5-min Apgar scores or in neonatal mortality. For the twin B, no significant differences were reported, but in 50 % of the studies information about the neonatal outcome of twin B was lacking.

**Table 2:** Overview results

Presentation of the twins		5-min Apgar scores <7				Neonatal mortality			
		No significant difference	Significant difference favouring vaginal delivery	Significant difference favouring caesarean delivery	Not reported	No significant difference	Significant difference favouring vaginal delivery	Significant difference favouring caesarean delivery	Not reported
Non-cephalic presentation twin A (8 studies [12–16, 23–25, 27])	Twin A	100 % (8 studies [12–16, 23–25, 27])	–	–	–	100 % (8 studies [12–16, 23–25, 27])	–	–	–
	Twin B	50 % (4 studies [12–16])	–	–	50 % (4 studies [23–25, 27])	50 % (4 studies [12–16])	–	–	50 % (4 studies [23–25, 27])
Non-cephalic presentation twin B (11 studies [9, 17–22, 24, 26–28])	Twin A	36 % (4 studies [9, 17, 18, 26])	–	–	64 % (7 studies [19–22, 24, 27, 28])	45 % (5 studies [9, 17, 18, 20, 21])	–	–	55 % (6 studies [19, 22, 24, 26–28])
	Twin B	82 % (9 studies [9, 17–21, 24, 26, 28])	–	9 % (1 study [22])	9 % (1 study[27])	82 % (9 studies [9, 17–22, 24, 27])	–	–	18 % (2 studies [26, 28])
Non-cephalic presentation twin A and B (1 study [11])	Twin A	100 % (1 study11)	–	–	–	100 % (1 study [11])	–	–	–
	Twin B	100 % (1 study [11])	–	–	–	100 % (1 study [11])	–	–	–

Significant differences were defined according to the definitions and statistics used in the different studies

^a^Essel [11]: only the abstract was available

^b^Blickstein [16]: only the abstract was available

**Table 3:** First twin in non-cephalic presentation

Author	Year	Study design	Mode of delivery		Gestational age (weeks)	Presentation		Birth weight		5-min Apgar score <7		Neonatal mortality	
			VD/CS	*N*		Twin A breech (%)	Twin B cephalic (%)	Twin A (g)	Twin B (g)	Twin A *N* (%)	Twin B *N* (%)	Twin A *N* (%)	Twin B *N* (%)
Sentilhes [12]	2007	Retr cohort	VD	124	37 ± 1	100 %	45 %	2,620 ± 363	2,555 ± 410	2 (2 %)	0	1 (1 %)	0
			CS	71	37 ± 1	100 %	52 %	2,762 ± 429	2,490 ± 446	2 (3 %)	1 (1 %)	0	1 (1 %)
										*p* > 0.05	*p* > 0.05	*p* > 0.05	*p* > 0.05
Griasaru [13]	2000	Retr cohort	VD	33	>32	100 %	52 %	2,636 ± 385	2,588 ± 456	0	0	0	0
			CS	38	>32	89 %	NR	2,589 ± 450	2,488 ± 475	0	0	0	0
Abu-Heija [14, 15]^a^	1998	Retr cohort	VD	42	37 ± 3	100 %	NR	2,566 ± 555	2,450 ± 482	NR	NR	3 (7 %)	1 (2 %)
			CS	87	38 ± 2	100 %	NR	2,712 ± 553	2,577 ± 594	NR	NR	2 (3 %)	0
										*p* > 0.05	*p* > 0.05	*p* > 0.05	*p* > 0.05
Blickstein [16]^b^	1993	Retr cohort	VD	24	NR^a^	100 %	100 %	NR^a^	NR^a^	NR	NR	NR	NR
			CS	35	NR^a^	100 %	100 %	NR^a^	NR^a^	NR	NR	NR	NR
										*p* > 0.05	*p* > 0.05	*p* > 0.05	*p* > 0.05
Nassar [23]	2004	Retr cohort	VD	35	36 ± 3	100 %	35 %	2,274 ± 486	NR	3 %	NR	6 %	NR
			CS	95	36 ± 3	100 %	45 %	2,344 ± 617	NR	15 %	NR	6 %	NR
										*p* > 0.05		*p* > 0.05	
Roopnarinesingh [24]	2002	Retr cohort	VD	18	>32	100 %	NR	1,560–2,960	NR	0	NR	0	NR
			CS	32	>32	100 %	NR	1,220–3,040	NR	0	NR	0	NR
Blickstein [25]	2000	Retr cohort	VD	53	36 ± 3	100 %	NR	2,454 ± 466	2,539 ± 547	7 (7 %)	NR	0	NR
		(Nullipara)	CS	156	36 ± 2	100 %	NR	2,527 ± 485	2,441 ± 533	16 (5 %)	NR	1 (0.3 %)	NR
										*p* > 0.05		*p* > 0.05	
		Retr cohort	VD	129	37 ± 2	100 %	49 %	2,609 ± 524	2,626 ± 519	14 (5 %)	NR	1 (0.4 %)	NR
		(Multipara)	CS	167	37 ± 3	100 %	44 %	2,662 ± 551	2,577 ± 568	17 (5 %)	NR	0	NR
										*p* > 0.05		*p* > 0.05	
Kelsick [27]	1982	Retr cohort	VD	194	NR	100 %	NR	2,000–4,000	2,000–4,000	NR	NR	2 (1 %)	NR
			CS	142	NR	100 %	NR	2,000–4,000	2,000–4,000	NR	NR	2 (1 %)	NR
												*p* > 0.05	

Significant differences were defined according to the definitions and statistics used in the different studies

*VD* Vaginal delivery, *CS* Caesarean section, *N* Number of twin pairs, *Retr* Retrospective, *NR* Not reported

^a^Abu-Heija [14, 15] did not report the percentage of 5-min Apgar scores <7, but did report the mean 5-min Apgar scores: 8 ± 1 in all groups (without significant differences)

^b^Blickstein [16]: only the abstract was available

### Twin B in non-cephalic presentation (Tables 2, 4)

Eleven studies including 2,166 twin pairs compared the mode of delivery of twins with twin B in non-cephalic presentation, including one high-quality randomized clinical trial [9] (60 twin pairs) and ten low-quality observational studies [17–22, 24, 26–28].

**Table 4:** Second twin in non-cephalic presentation

Author	Year	Study design	Mode of delivery		Gestational age (weeks)	Presentation		Birth weight		5-min Apgar score <7		Neonatal mortality	
			VD/CS	*N*		Twin A cephalic (%)	Twin B breech (%)	Twin A (g)	Twin B (g)	Twin A *N* (%)	Twin B *N* (%)	Twin A *N* (%)	Twin B *N* (%)
Differences in neonatal outcome													
Atis [22]^a^	2011	Retr cohort	VD	289	36 ± 6	100 %	NR	NR	2,335 ± 443	NR	44 (15 %)	NR	2 (1 %)
			CS	193	36 ± 6	100 %	NR	NR	2,558 ± 648	NR	16 (8 %)	NR	1 (1 %)
											*p* < 0.05		*p* > 0.05
No differences in neonatal outcome													
Rabinovici [9]	1986	RCT	VD	33	38 ± 2	100 %	61 %	2,477 ± 370	2,459 ± 510	0	1 (3 %)	0	0
			CS	27	38 ± 2	100 %	67 %	2,533 ± 423	2484 ± 632	1 (4 %)	1 (4 %)	0	0
											*p* > 0.05		
Wells [17]	1991	Retr cohort	VD-breech extraction	42	37	100 %	100 %	2,660	2,537	0	1 (2 %)	0	0
			VD-external version	11	35	100 %	100 %	2,363	2,389	0	0	0	0
			CS	29	37	100 %	100 %	2,701	2,521	0	0	0	0
										*p* > 0.05	*p* > 0.05		
Gocke [18]	1989	Retr cohort	VD-breech extraction	55	37	100 %	100 %	2,544	2,569	0	1 (3 %)	0	0
			VD-exernal version	41	36	100 %	100 %	2,399	2,365	0	0	0	0
			CS	40	36	100 %	100 %	2,356	2,347	0	0	0	0
											*p* > 0.05		
Caukwell [19]^a^	2002	Retr cohort	VD	64	≥37	NR	NR	NR	NR	NR	4 (6 %)	NR	0
			CS	34	≥37	NR	NR	NR	NR	NR	3 (9 %)	NR	0
											*p* > 0.05		
Winn [20]^b^	2001	Retr cohort	VD	31	34 ± 2	NR	100 %	NR	2,115 ± 415	NR	NR	0	0
			CS-without labour	34	35 ± 2	NR	100 %	NR	2,242 ± 456	NR	NR	0	0
			CS-with labour	36	34 ± 2	NR	100 %	NR	2,215 ± 442	NR	NR	0	0
											*p* > 0.05		
Acker [21]^c^	1981	Retr cohort	VD	76	NR	NR	100 %	>1,500	>1,500	NR	11 (15 %)	0	0
			CS	75	NR	NR	NR	>1,500	>1,500	NR	7 (10 %)	0	0
											*p* > 0.05		
Roopnarinesingh [24]	2002	Retr cohort	VD	54	>32	NR	100 %	NR	1,560–2,960	NR	0	NR	0
			CS	33	>32	NR	100 %	NR	1,220–3,040	NR	0	NR	1 (3 %)
Mauldin [26]^a^	1998	Prosp cohort	VD-breech extraction	41	35 ± 4	NR	NR	2,270 ± 741	2,167 ± 728	14	17	NR	NR
			VD-external version	19	34 ± 2	NR	NR	2,233 ± 561	2,295 ± 702	0	10	NR	NR
			CS	24	35 ± 4	NR	NR	2,169 ± 680	2,116 ± 739	16	20	NR	NR
										*p* > 0.05	*p* > 0.05		
Kelsick [27]	1982	Retr cohort	VD	590	NR	NR	100 %	2,000–4,000	2,000–4,000	NR	NR	NR	1 (0.2 %)
			CS	141	NR	NR	100 %	2,000–4,000	2,000–4000	NR	NR	NR	1 (0.2 %)
													*p* > 0.05
Greig [28]^a,d^	1992	Retr cohort	VD	12	NR	NR	NR	NR	1,500–1,999	NR	NR	NR	NR
		(1,500–1,999 g)	CS	24	NR	NR	NR	NR	1,500–1,999	NR	NR	NR	NR
											*p* > 0.05		
		Retr cohort	VD	21	NR	NR	NR	NR	1,500–1,999	NR	NR	NR	NR
		(2,000–2,499 g)	CS	31	NR	NR	NR	NR	1,500–1,999	NR	NR	NR	NR
											*p* > 0.05		
		Retr cohort	VD	21	NR	NR	NR	NR	1,500–1,999	NR	NR	NR	NR
		(≥2,500 g)	CS	46	NR	NR	NR	NR	1,500–1,999	NR	NR	NR	NR
											*p* > 0.05		

Significant differences were defined according to the definitions and statistics used in the different studies

*VD* Vaginal delivery, *CS* Caesarean section, *N* Number of twin pairs, *RCT* Randomized controlled trial, *Retr* Retrospective, *Prosp* Prospective, *NR* Not reported

^a^Atis [22], Caukwell [19], Mauldin [26], Greig [28]: twin B in non-cephalic postition, not further specified to breech or transverse position

^b^Winn [20] did not report the percentage of 5-min Apgar scores <7, but did report the mean 5-min Apgar scores: 8 ± 1 in VD and CS-with labour group, and 9 ± 1 in the CS-without labour group (without significant differences)

^c^Acker [21]: twins delivered by caesarean section: twin A or B was in non-cephalic presentation

^d^Greig [28] did not report the percentage of 5-min Apgar scores <7, but did report the mean 5-min Apgar scores: 1,500–1,999 g and 2,000–2,499 g: 9 in the VD and 8 in the CS group (without significant differences); ≥2,500 g: 9 in the VD group and 9 in the CS group (without significant differences)

The randomized clinical trial that compared vaginal with caesarean delivery did not report a significant difference in low 5-min Apgar scores or in neonatal mortality for the twin A and B [9].

For the twin A, none of the studies did report significant differences in neonatal outcome but information about the neonatal outcome of twin A was lacking in 64 % (low 5-min Apgar scores) and 55 % (neonatal mortality) of the studies.

For the twin B, most studies (82 %) did not report a significant difference in low 5-min Apgar scores or neonatal mortality but one study [22] (482 twin pairs) did report a significant difference in low 5-min Apgar scores favouring caesarean delivery (*p* < 0.05). This study [22] did not report a significant difference in neonatal mortality.

### Both twins in non-cephalic presentation (Tables 2, 5)

One moderate-quality observational study [11] including 68 twin pairs compared the mode of delivery of twins with both twins in non-cephalic presentation. No significant differences were reported for twins A and B.

**Table 5:** Both twins in non-cephalic presentation

Author	Year	Study design	Mode of delivery		Gestational age (weeks)	Presentation		Birth weight		5-min Apgar score <7		Neonatal mortality	
			VD/CS	*N*		Twin A breech (%)	Twin B breech (%)	Twin A (g)	Twin B (g)	Twin A *N* (%)	Twin B *N* (%)	Twin A *N* (%)	Twin B *N* (%)
Essel [11]	1996	Prosp cohort	VD	41	NR	100 %	85 %	NR^a^	NR^a^	NR^a^	NR^a^	NR^a^	NR^a^
			SC	27	NR	100 %	93 %	NR^a^	NR^a^	NR^a^	NR^a^	NR^a^	NR^a^
										*p* > 0.05	*p* > 0.05	*p* > 0.05	*p* > 0.05

Significant differences were defined according to the definitions and statistics used in the different studies

*VD* Vaginal delivery, *CS* Caesarean section, *N* Number 1 of twin pairs, *Prosp* Prospective, *NR* Not reported

^a^Essel [11]: only the abstract was available

## Discussion

The aim of the current review was to compare vaginal with caesarean delivery for twin deliveries with twin A in non-cephalic presentation, twin B in non-cephalic presentation and both twins in non-cephalic presentation. This evaluation is important because of the increasing numbers of caesarean sections without adequate supporting evidence for their use [8].

One high-quality clinical trial [9] (60 twin pairs) and 16 moderate/low-quality observational studies [11–21, 23–28] (3,167 twin pairs) showed no difference in neonatal outcome between vaginal and caesarean deliveries in twin A and/or B. Only one low-quality observational study [22] (482 twin pairs) reported a significant difference in low 5-min Apgar scores favouring caesarean delivery but there was no significant difference in neonatal mortality.

A reason to recommend caesarean over vaginal delivery if twin A is presenting non-cephalically might be to avoid the possibility of interlocking twins, which theoretically could occur in breech/cephalic and breech/transverse presenting twins. However, the incidence of interlocked twins is very low [1]. Furthermore, according to Hannah et al. [29] in term breech singletons, planned caesarean section is better than vaginal delivery. However, a previous Cochrane review did describe the maternal and neonatal outcome of the same clinical trial [9] we cited, and they stated that caesarean delivery of a non cephalic presenting twin B is associated with increased maternal morbidity but not with improved neonatal outcome, and that a policy of caesarean delivery should not be adopted without further controlled trials [30]. Additionally, previous research did not find excessive morbidity or mortality associated with vaginal delivery of non-cephalic presenting twins compared with cephalic presenting twins [31–35]. Because we include only studies that compared non-cephalic presenting twins with each other, these reports were excluded.

A few studies provided detailed information about the mode of vaginal delivery like external cephalic version or (assisted) breech extraction. Both external version [36–38] and breech extraction [39–41] are recommended in the literature. To our knowledge, there are no randomized controlled data comparing external version with breech extraction. Future research about this subject might be useful.

A limitation of this review is that the included studies had relatively small sample sizes. However, in a meta-analysis from 2003, Hodge et al. [2] pooled the data of four studies that we described separately [9, 13, 17, 25]. They remarked that even the sample size of the pooled data was too small to draw conclusions. Therefore, although after including more recent studies statistic evidence for the best mode of delivery for twins presenting non-cephalically is still missing and no strong recommendation can be made. Furthermore, most studies did not correct (statistically or by randomisation) for confounding factors. Important confounding factors are parity or medical, obstetric or emergency indications for a caesarean section.

Additionally, most studies did not provide information about monoamnioticity. Therefore, it is mostly unknown if only diamniotic twins were included, or if monoamniotic and diamniotic twins were mixed. Ideally, you should analyse these groups separately. However, the bias due to this cause might be limited if the percentage of monoamniotic twins is equal in both the vaginal and the caesarean delivery group.

Finally, in two studies [11, 16], we used information from the abstract only because we were not able to get full text of both papers. However, we were able to retrieve all information we needed from the abstract, but ideally studies should be assessed with the full text available.

Therefore, our results have to be interpreted with caution.

## Conclusion

Our results do not suggest benefit of caesarean over vaginal delivery for selected twin gestations with twin A and/or twin B in non-cephalic presentation. However, no final conclusion can be drawn. Randomized studies with sufficient power are required to make a strong recommendation.

## Appendix 1

Medline: (((((twins[Title/Abstract] OR twin[Title/Abstract]) OR sibling[Title/Abstract]) OR siblings[Title/Abstract]) OR reciprocal[Title/Abstract]) OR reciprocals[Title/Abstract])) AND (((((((((breech presentation[Title/Abstract] OR breech-presentation[Title/Abstract]) OR breech-presentations[Title/Abstract]) OR breech[Title/Abstract]) OR non-vertex[Title/Abstract]) OR non-vertex-presentation[Title/Abstract]) OR non-vertex-presentations[Title/Abstract]) OR non-cephalic[Title/Abstract]) OR non-cephalic-presentation[Title/Abstract]) OR non-cephalic-presentations[Title/Abstract])) AND ((((((((((((((((((((((((((vaginal delivery[Title/Abstract] OR vaginal[Title/Abstract]) OR vaginally[Title/Abstract]) OR deliver[Title/Abstract]) OR delivered[Title/Abstract]) OR delivery[Title/Abstract]) OR deliveries[Title/Abstract]) OR childbirth[Title/Abstract]) OR childbirths[Title/Abstract]) OR accouchement[Title/Abstract]) OR bearing[Title/Abstract]) OR birth[Title/Abstract]) OR births[Title/Abstract]) OR birthing[Title/Abstract]) OR bringing forth[Title/Abstract]) OR childbearing[Title/Abstract]) OR confinement[Title/Abstract]) OR geniture[Title/Abstract]) OR labor[Title/Abstract]) OR labour[Title/Abstract]) OR lying-in[Title/Abstract]) OR paturition[Title/Abstract]) OR parturitions[Title/Abstract]) OR travail[Title/Abstract]) OR extraction[Title/Abstract]) OR extractions[Title/Abstract]) OR (((((((caesarian section[Title/Abstract] OR caesarian sections[Title/Abstract]) OR caesarian[Title/Abstract]) OR caesarian[Title/Abstract]) OR section[Title/Abstract]) OR sections[Title/Abstract]) OR abdominal[Title/Abstract]) OR abdominally[Title/Abstract])).

Embase: (((((twins:ab,ti OR twin:ab,ti) OR sibling:ab,ti) OR siblings:ab,ti) OR reciprocal:ab,ti) OR reciprocals:ab,ti) AND (((((((((breech presentation:ab,ti OR breech-presentation:ab,ti) OR breech-presentations:ab,ti) OR breech:ab,ti) OR non-vertex:ab,ti) OR non-vertex-presentation:ab,ti) OR non-vertex-presentations:ab,ti) OR non-cephalic:ab,ti) OR non-cephalic-presentation:ab,ti) OR non-cephalic-presentations:ab,ti) AND ((((((((((((((((((((((((((vaginal delivery:ab,ti OR vaginal:ab,ti) OR vaginally:ab,ti) OR deliver:ab,ti) OR delivered:ab,ti) OR delivery:ab,ti) OR deliveries:ab,ti) OR childbirth:ab,ti) OR childbirths:ab,ti) OR accouchement:ab,ti) OR bearing:ab,ti) OR birth:ab,ti) OR births:ab,ti) OR birthing:ab,ti) OR bringing forth:ab,ti) OR childbearing:ab,ti) OR confinement:ab,ti) OR geniture:ab,ti) OR labor:ab,ti) OR labour:ab,ti) OR lying-in:ab,ti) OR paturition:ab,ti) OR parturitions:ab,ti) OR travail:ab,ti) OR extraction:ab,ti) OR extractions:ab,ti) OR (((((((caesarian section:ab,ti OR caesarian sections:ab,ti) OR caesarian:ab,ti) OR caesarian:ab,ti) OR section:ab,ti) OR sections:ab,ti) OR abdominal:ab,ti) OR abdominally:ab,ti)).

Cochrane: (Twin OR twins OR sibling OR siblings OR reciprocal OR reciprocals) AND (breech-presentation OR breech-presentation OR breech OR non-vertex OR non-cephalic) AND (vaginal OR vaginally or deliver OR delivered OR delivery OR deliveries OR childbirth OR childbirths OR accouchement OR bearing OR birth OR births OR birthing OR brining forth OR childbearing OR confinement OR geniture OR labor OR labour OR caesarian OR caesarians OR section OR sections OR abdominal OR abdominally) Field: abstract.

CINAHL: (Twin OR twins OR sibling OR siblings OR reciprocal OR reciprocals) AND (breech-presentation OR breech-presentation OR breech OR non-vertex OR non-cephalic) AND (vaginal OR vaginally or deliver OR delivered OR delivery OR deliveries OR childbirth OR childbirhs OR accouchement OR bearing OR birth OR births OR birthing OR brining forth OR childbearing OR confinement OR geniture OR labor OR labour OR caesarian OR caesarians OR section OR sections OR abdominal OR abdominally) Field: abstract.

## References

[1] Cruikshank DP (2007). Intrapartum management of twin gestations. Obstet Gynecol.

[2] Hogle KL, Hutton EK, McBrien KA (2003). Cesarean delivery for twins: a systematic review and meta-analysis. Am J Obstet Gynecol.

[3] Stichting Perinatale Registratie Nederland, Perinatale Zorg in Nederland 2008. Utrecht: Stichting Perinatale Registratie nederland. (2011)

[4] Pope RJ, Weintraub AY, Sheiner E (2010). Vaginal delivery of vertex–nonvertex twins: a fading skill?. Arch Gynecol Obstet.

[5] Boggess KA, Chisholm CA (1997). Delivery of the nonvertex second twin: a review of the literature. Obstet Gynecol Surv.

[6] Dutch Society for Obstetrics and Gynecology (2011) Guideline Multiple Gestation version 2.0. March 2005. Available at http://www.nvog.nl

[7] American College of Obstetricians and Gynecologsits Committee on Practice Bulletins-Obstetrics et al (2004) ACOG Practice Bulletin #56: multiple gestation: complicated twin, triplet, and high-order multifetal pregnancy. Obstet Gynecol 104(4):869–883 10.1097/00006250-200410000-00046 15458915

[8] Carroll MA, Yeomans ER (2006). Vaginal delivery of twins. Clin Obstet Gynecol.

[9] Rabinovici J, Barkai G, Reichman B (1987). Randomized management of the second nonvertex twin: vaginal delivery or cesarean section. Am J Obstet Gynecol.

[10] Higgins JPT, Green S (2011) Cochrane handbook for Systematic Reviews of Interventions Version 5.1.0. The Cochrane Collaboration, 2011. Available at http://www.cohrane-handbook.org

[11] Essel JK, Opai-Tetteh ET (1996). Is routine caesarean section necessary for breech-breech and breech-transverse twin gestations?. S Afr Med J.

[12] Sentilhes L, Goffinet F, Talbot A (2007). Attempted vaginal versus planned cesarean delivery in 195 breech first twin pregnancies. Acta Obstet Gynecol Scand.

[13] Grisaru D, Fuchs S, Kupferminc MJ (2000). Outcome of 306 twin deliveries according to first twin presentation and method of delivery. Am J Perinatol.

[14] Bu-Heija AT, Ziadeh S, Obeidat A (1998). Mode of delivery and perinatal results of the breech first twin. J Obstet Gynaecol.

[15] Bu-Heija AT, Ziadeh S, Abukteish F (1998). Retrospective study of outcome on vaginal and abdominal delivery in twin pregnancy in which twin 1 is presenting by the breech. Arch Gynecol Obstet.

[16] Blickstein I, Weissman A, Ben-Hur H (1993). Vaginal delivery of breech-vertex twins. J Reprod Med.

[17] Wells SR, Thorp JM, Bowes WA (1991). Management of the nonvertex second twin. Surg Gynecol Obstet.

[18] Gocke SE, Nageotte MP, Garite T (1989). Management of the nonvertex second twin: primary cesarean section, external version, or primary breech extraction. Am J Obstet Gynecol.

[19] Caukwell S, Murphy DJ (2002). The effect of mode of delivery and gestational age on neonatal outcome of the non-cephalic- presenting second twin. Am J Obstet Gynecol.

[20] Winn HN, Cimino J, Powers J (2001). Intrapartum management of nonvertex second-born twins: a critical analysis. Am J Obstet Gynecol.

[21] Acker D, Lieberman M, Holbrook RH (1982). Delivery of the second twin. Obstet Gynecol.

[22] Atis A, Aydin Y, Donmez M (2011). Apgar scores in assessing morbidity of the second neonate of cephalic/non-cephalic twins in different delivery modes. J Obstet Gynaecol.

[23] Nassar AH, Maarouf HH, Hobeika EM (2004). Breech presenting twin A: is vaginal delivery safe?. J Perinat Med.

[24] Roopnarinesingh AJ, Sirjusingh A, Bassaw B (2002). Vaginal breech delivery and perinatal mortality in twins. J Obstet Gynaecol.

[25] Blickstein I, Goldman RD, Kupferminc M (2000). Delivery of breech first twins: a multicenter retrospective study. Obstet Gynecol.

[26] Mauldin JG, Newman RB, Mauldin PD (1998). Cost-effective delivery management of the vertex and nonvertex twin gestation. Am J Obstet Gynecol.

[27] Kelsick F, Minkoff H (1982). Management of the breech second twin. Am J Obstet Gynecol.

[28] Greig PC, Veille JC, Morgan T (1992). The effect of presentation and mode of delivery on neonatal outcome in the second twin. Am J Obstet Gynecol.

[29] Hannah ME, Hannah WJ, Hewson SA, Term Breech Trial Collaborative Group (2000). Planned caesarean section versus planned vaginal birth for breech presentation at term: a randomised multicentre trial. Lancet.

[30] Crowther CA (2000) Caesarean delivery for the second twin. Cochrane Database Syst Rev:CD000047 10.1002/14651858.CD000047 10796103

[31] Fishman A, Grubb DK, Kovacs BW (1993). Vaginal delivery of the nonvertex second twin. Am J Obstet Gynecol.

[32] Mazor M, Leiberman JR, Dreval D (1986). Management and outcome of vertex-breech and vertex-vertex presentation in twin gestation: a comparative study. Eur J Obstet Gynecol Reprod Biol.

[33] Blickstein I, Schwartz-Shoham Z, Lancet M (1987). Vaginal delivery of the second twin in breech presentation. Obstet Gynecol.

[34] Sibony O, Touitou S, Luton D (2006). Modes of delivery of first and second twins as a function of their presentation. Study of 614 consecutive patients from 1992 to 2000. Eur J Obstet Gynecol Reprod Biol.

[35] Oettinger M, Ophir E, Markovitz J (1993). Is cesarean section necessary for delivery of a breech first twin?. Gynecol Obstet Invest.

[36] Chervenak FA, Johnson RE, Berkowitz RL (1983). Intrapartum external version of the second twin. Obstet Gynecol.

[37] Tchabo JG, Tomai T (1992). Selected intrapartum external cephalic version of the second twin. Obstet Gynecol.

[38] Kaplan B, Peled Y, Rabinerson D (1995). Successful external version of B-twin after the birth of A-twin for vertex–non-vertex twins. Eur J Obstet Gynecol Reprod Biol.

[39] Berglund L, Axelsson O (1989). Breech extraction versus cesarean section for the remaining second twin. Acta Obstet Gynecol Scand.

[40] Smith SJ, Zebrowitz J, Latta RA (1997). Method of delivery of the nonvertex second twin: a community hospital experience. J Matern Fetal Med.

[41] Chauhan SP, Roberts WE, McLaren RA (1995). Delivery of the nonvertex second twin: breech extraction versus external cephalic version. Am J Obstet Gynecol.

